# Concurrent urachal abscess and florid cystitis glandularis masquerading as malignancy: a case report and literature review

**DOI:** 10.1186/s12893-021-01430-w

**Published:** 2022-03-21

**Authors:** Yi-Hsuan Chen, Jen-Shu Tseng

**Affiliations:** Department of Urology, Mackay Memorial Hospital, No. 92, Sec. 2, Zhongshan N. Rd., Zhongshan Dist., 104 Taipei, Taiwan

**Keywords:** Urachus, Abscess, Bladder neoplasm, Cystitis glandularis

## Abstract

**Background:**

The urachus is the embryological remnant of the cloaca and allantois. Failure of its regression can cause diseases any time after birth. It is difficult to differentiate an abscess from urachal adenocarcinoma based on the clinical presentation and image findings. Cystitis glandularis reflects chronic irritation of the bladder urothelium, and tumor-like florid cystitis glandularis can be misdiagnosed as malignancy. We report a patient with concurrent urachal abscess and florid cystitis glandularis which increased the resemblance of malignancy.

**Case presentation:**

A 57-year-old female was incidentally found to have a heterogeneous pelvic mass abutting the urinary bladder. A cystoscopy examination revealed protruding tumors located in the bladder dome. Her blood test results were all normal, and urinalysis showed microscopic hematuria. Urachal cancer was diagnosed and en bloc excision of the umbilicus, tumor, and the involved bladder dome was performed. Pathology revealed urachal abscess with concurrent cystitis glandularis within the urinary bladder. No malignancy was identified in the resected specimen.

**Conclusions:**

It is challenging to distinguish urachal abscess from a malignant tumor based on the clinical presentation and imaging studies. As in our case, the coexistence of urachal abscess and tumor-like florid cystitis glandularis increased the resemblance to a malignancy. This is the first reported case of the concurrence of these two disease entities, and emphasizes that the detection of bladder tumors on cystoscopy is not sufficient to make the diagnosis of urachal cancer with bladder involvement.

## Background

During embryogenesis, the allantois and cloaca give rise to the urachus, which then involutes and obliterates into the median umbilical ligament [1]. Failure of this process can lead to pathological conditions and complications later in life. Infection and malignancy arising from urachal remnants are the main complications in adults [2]. Urachal abscess and carcinoma share overlapping presentations and imaging features, and cases of one entity mimicking the other have been reported [3, 4].

Cystitis glandularis is a proliferative disorder of bladder epithelium, which may be caused by normal urothelium reacting to chronic irritation such as infection or malignancy [5].

Herein, we report a patient who was initially diagnosed and treated for urachal cancer with bladder invasion. Pathology later revealed urachal abscess and cystitis glandularis. We also review the clinical presentations, imaging features, and management of urachal mass and cystitis glandularis.

## Case presentation

**Fig. 1:**
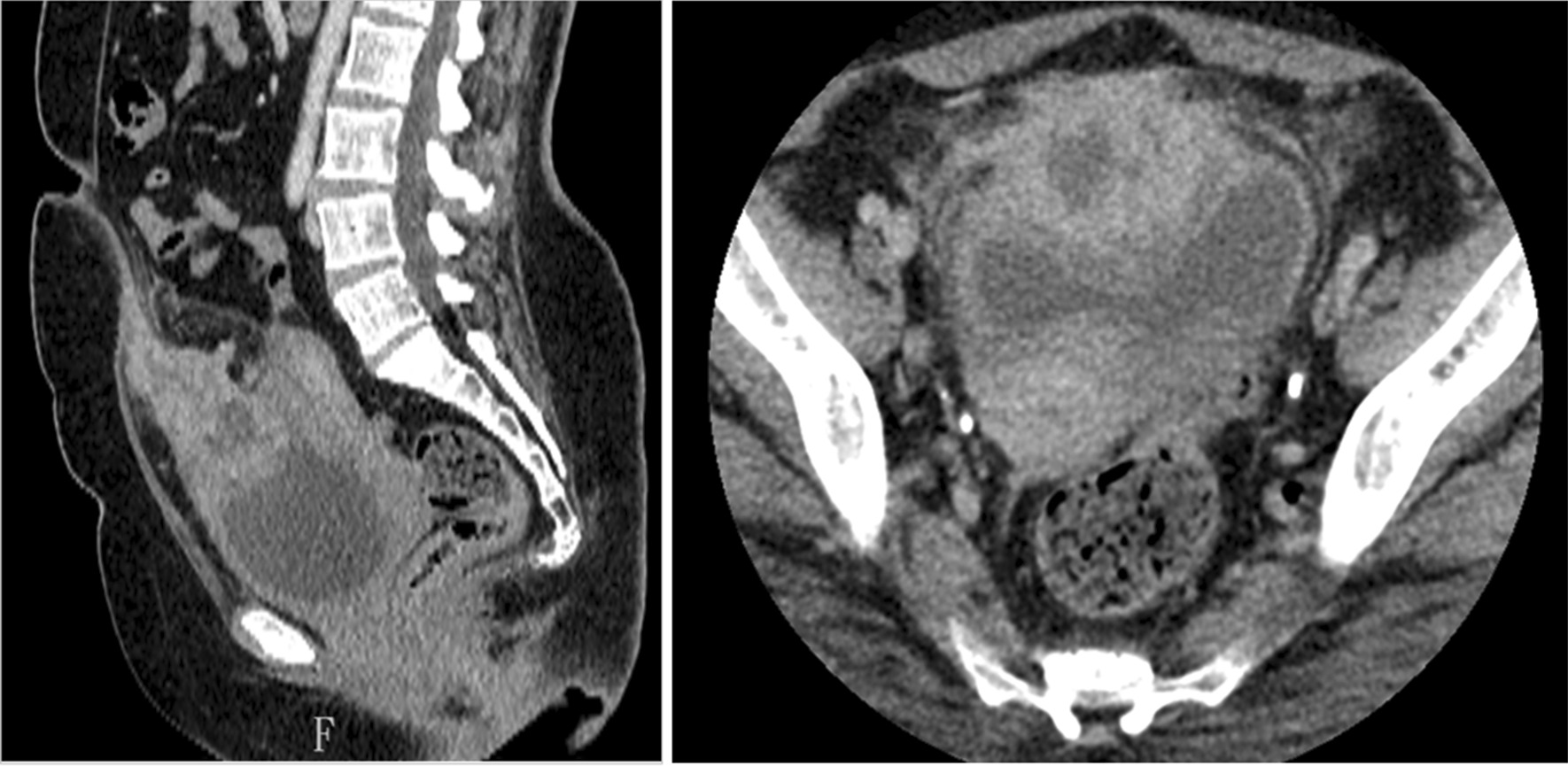
Abdominal CT showing a midline suprapubic mass of heterogeneous enhancement between umbilicus and bladder. The mass consisted of low attenuation cystic element surrounded by thick, enhancing wall

A 57-year-old female was incidentally diagnosed with an ovarian cyst during a clinic visit for occasional postmenopausal vaginal spotting, and was referred to our hospital. She denied fever, abdominal pain, lower urinary tract symptoms, hematuria, or any other symptoms. Transvaginal ultrasound showed a 6-cm heterogeneous pelvic mass with mixed echogenicity located anterior to the uterus. Her uterus, endometrium, and bilateral adnexa were unremarkable. Contrast-enhanced computed tomography (CT) showed an enhancing pelvic mass with solid and cystic components closely abutting the bladder dome and abdominal wall with adjacent fat stranding. No conspicuous local invasion, enlarged pelvic lymph nodes, or distant metastases were noted (Fig. 1).

**Fig. 2:**
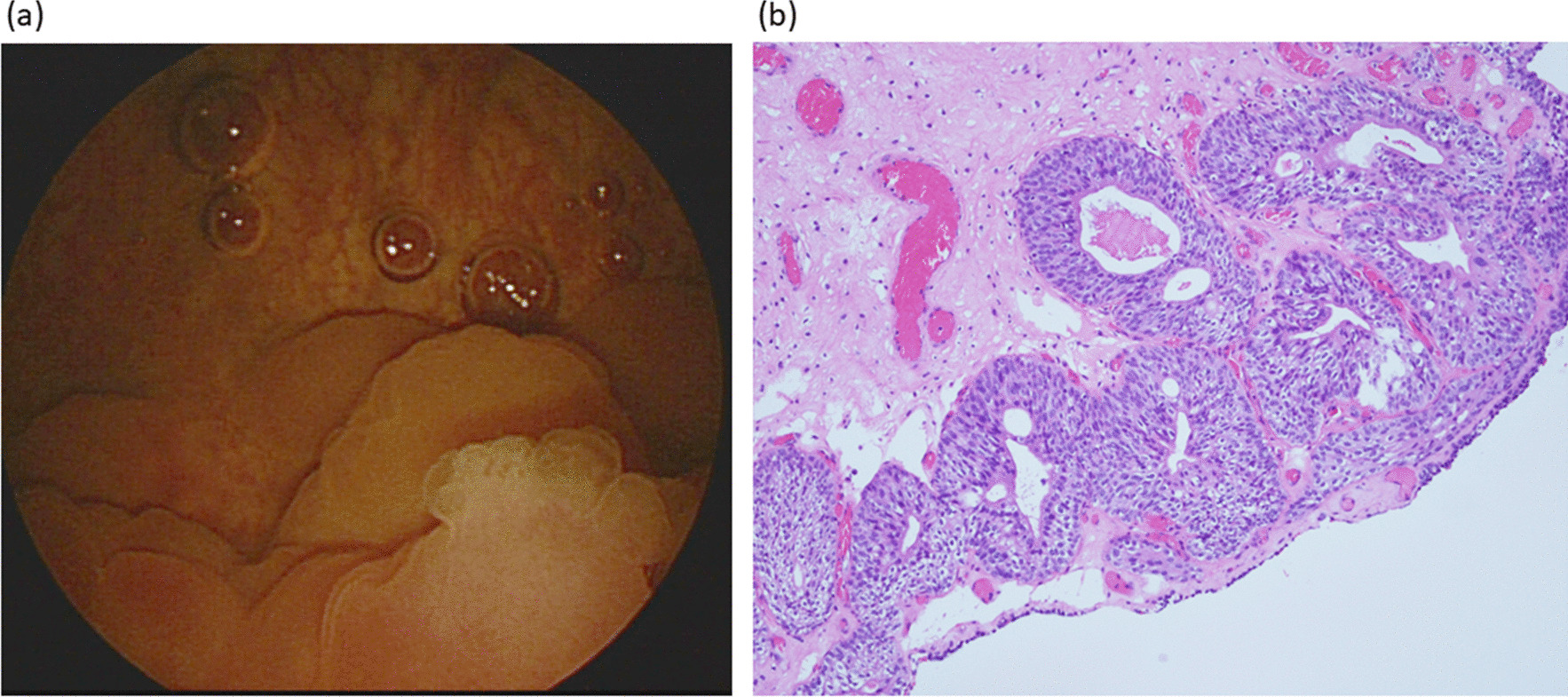
**a** Cystoscopy demonstrated protruding tumors in bladder dome. Normal mucosa was observed in its periphery. **b** Pathology finding of the bladder tumors showed layers of columnar epithelium surrounding dilated lumens located in submucosa, indicating cystitis glandularis

Cystoscopy revealed protruding tumors in the bladder dome (Fig. 2a). Complete blood count, serum biochemistry, and tumor markers including carcinoembryonic antigen and carbohydrate antigen 125 were normal. Urinalysis showed microscopic hematuria.

As a malignant tumor of the urachus was suspected, the patient underwent open surgical en bloc excision of the umbilicus, tumor, and involved bladder dome via a lower midline incision. A piece of thickened omentum adhered to the tumor was found intraoperatively, and was removed wholly due to the suspicion of omental cake. Pathology revealed a 6-cm necrotic mass with abscess formation and the focal presence of urachal remnants. Cystitis glandularis was identified in the resected bladder (Fig. 2b). Cytokeratin staining confirmed the absence of malignancy. The postoperative course was smooth, and follow-up abdominal CT 3 months later revealed complete resolution without recurrence.

## Discussion and conclusions

Infection of urachal remnants occurs most commonly in children and young adults [6]. The route of infection may be lymphatic, hematogenous, or from the adjacent urinary bladder. Various organisms have been identified from bacterial cultures [7], however abscess formation is rare and generally occurs in severe infections [6]. The clinical presentation of infected urachal remnants is often insidious and can go unrecognized for years. General signs such as leukocytosis or fever can be absent, and urinalysis or urine cultures are usually normal [6]. Our patient remained asymptomatic, and the disease was only found on ultrasound and CT.

Malignant urachal neoplasms comprise less than 0.5% of all bladder malignancies and are predominantly adenocarcinoma, even though the urachus is commonly lined by urothelium. Several studies have reported that chronic irritation-induced metaplasia followed by malignant transformation may explain the pathophysiology of urachal cancer [2, 7]. Unlike infection, malignant degeneration of the urachus generally occurs in middle or older age, and it has a poor prognosis compared to primary bladder adenocarcinoma [6].

About 90% of urachal cancers develop in the juxtavesical portion of the urachus and may extend toward the umbilicus and bladder [7]. Over 70% of patients present with hematuria, and other symptoms include lower abdominal mass, abdominal pain, lower urinary tract symptoms, and discharge from the umbilicus [2]. Due to its silent extraperitoneal nature, local invasion or metastasis is common at diagnosis. Metastases to pelvic lymph nodes, omentum, lungs, liver, and brain have also been reported [4].

As in our patient, urachal masses are often discovered incidentally in imaging studies. However, abscesses and malignancies are often indistinguishable in most imaging modalities [3, 4]. Ultrasonography demonstrates a midline mass in continuity with the bladder with complex heterogeneous echogenicity. In CT, either urachal abscess or malignancy may appear as a heterogeneously enhanced mass with solid, cystic, or a combination of both components. Low attenuation areas inside can represent infected abscess fluid or mucin content of carcinoma [2, 7].

Some imaging features have been described that can be used to differentiate a urachal mass. For example, calcification occurs in 50–70% of malignancies but rarely in abscesses. In addition, bladder wall involvement or a lack of adjacent inflammation also suggests malignant disease [7]. Percutaneous needle aspiration or cystoscopic biopsy may help to establish a definite diagnosis of a urachal mass [4].

The management of urachal abscess in adults usually includes broad-spectrum antibiotics and drainage followed by surgical excision. A 30% reinfection rate of residual urachal remnants has been reported, and there is also a theoretical risk of malignant degeneration [6]. Radical or partial cystectomy with en bloc excision of urachal tissues and the umbilicus is often required when treating urachal carcinoma with curative intent [2].

Cystitis glandularis is characterized by urothelium invaginating into the underlying lamina propria (Von Brunn’s nests) and foci of cuboidal or columnar epithelium with glandular metaplasia. Chronic inflammation of the bladder caused by infection, urolithiasis, outlet obstruction, and indwelling catheter has been implicated in the pathogenesis [8]. Cystitis glandularis has also been reported to coexist with adenocarcinoma of the bladder or urachus [5]. Although it has been hypothesized to be a potential precursor of adenocarcinoma, the risk of developing malignancy is still controversial, and there is currently insufficient evidence as to whether cystitis glandularis should be regarded as a premalignant lesion [9].

Patients often complain of urgency, frequency, dysuria, and sometimes hematuria. The trigone and bladder neck are commonly affected, which may lead to obstructive symptoms or hydronephrosis [10]. As mentioned, florid cystitis glandularis may resemble other neoplasms of the bladder, and a cystoscopic biopsy is sometimes necessary for differentiation. Treatment options include elimination of irritation, intravesical instillation, oral anti-inflammatory agents, transurethral resection, and even cystectomy with urinary diversion [8].

A bladder infection may result in both urachal remnant infection with subsequent abscess formation and cystitis glandularis in chronic settings. To the best of our knowledge, our patient is the first reported case to have both of these diseases concurrently.

In conclusion, urachal abscess is a rare condition in adults. It is challenging to distinguish urachal abscess from a malignant tumor based on clinical presentation and imaging studies. As in our case, the coexistence of urachal abscess and tumor-like florid cystitis glandularis is possible, which increases the difficulty in making a correct diagnosis. This combination of diseases should be taken into consideration as one of the differential diagnoses. This case also emphasizes that the detection of bladder tumors on cystoscopy is not sufficient to make the diagnosis of urachal cancer with bladder involvement.

## Data Availability

The datasets used and analyzed during the current study are available from the corresponding author on reasonable request.

## Abbreviations

CT: Computed tomography
